# The function of the co-chaperone ERdj4 in diverse (patho-)physiological conditions

**DOI:** 10.1007/s00018-021-04082-4

**Published:** 2021-12-24

**Authors:** Lea Daverkausen-Fischer, Felicitas Pröls

**Affiliations:** Faculty of Medicine, Institute of Anatomy II, University of Cologne, Joseph-Stelzmann-Str. 9, 50931 Cologne, Germany

**Keywords:** UPR, ERAD, BiP/GRP78, HSP70, Diabetes, Cell differentiation

## Abstract

Accumulation of misfolded proteins in the endoplasmic reticulum (ER) induces a well-orchestrated cellular response to reduce the protein burden within the ER. This unfolded protein response (UPR) is controlled primarily by three transmembrane proteins, IRE1α, ATF6, and PERK, the activity of which is controlled by BiP, the ER-resident Hsp70 protein. Binding of BiP to co-chaperones via their highly conserved J-domains stimulates the intrinsic ATPase activity of BiP, thereby providing the energy necessary for (re-)folding of proteins, or for targeting of misfolded proteins to the degradation pathway, processes specified and controlled by the respective co-chaperone. In this review, our aim is to elucidate the function of the co-chaperone ERDJ4, also known as MDG1, MDJ7, or DNAJB9. Knockout and knockin experiments clearly point to the central role of ERDJ4 in controlling lipogenesis and protein synthesis by promoting degradation of SREBP1c and the assembly of the protein complex mTORC2. Accumulating data reveal that ERDJ4 controls epithelial-to-mesenchymal transition, a central process during embryogenesis, in wound healing, and tumor development. Overexpression of ERdj4 has been shown to improve engraftment of transplanted human stem cells, possibly due to its ability to promote cellular survival in stressed cells. High ERDJ4-plasma levels are specific for fibrillary glomerulonephritis and serve as a diagnostic marker. As outlined in this review, the functions of ERDJ4 are manifold, depending on the cellular (patho-) physiological state, the cellular protein repertoire, and the subcellular localization of ERDJ4.

## Introduction

**Fig. 1:**
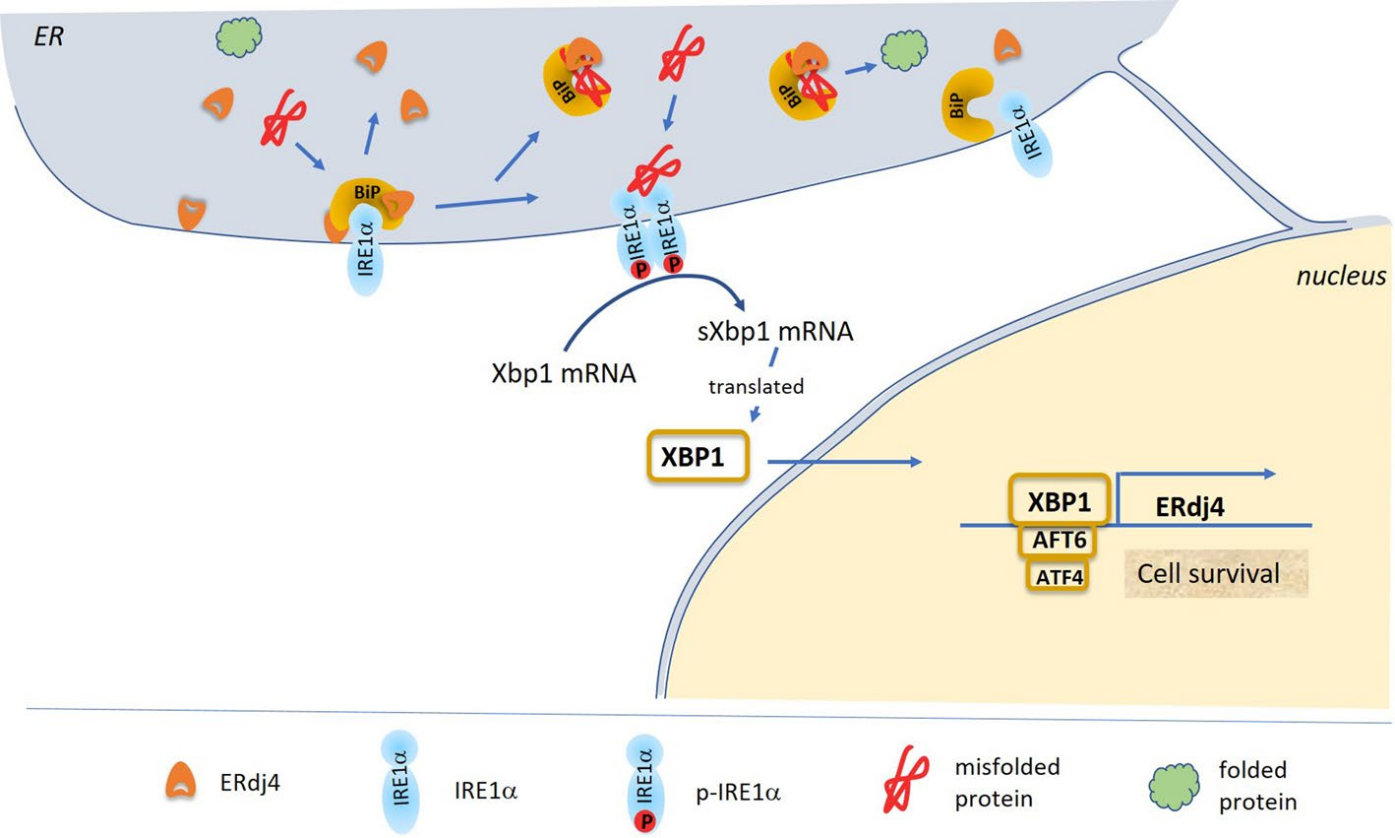
Schematic illustration of the ERDJ4-mediated ER-stress response. Under control conditions, the IRE1α/BiP complex is stabilized by ERDJ4, thereby inhibiting the IRE1α-induced signaling pathway. When increasing amounts of misfolded protein accumulate in the ER, ERDJ4 binds to it to transport it to BiP for initiation of its refolding (ER stress). Upon dissociation of ERDJ4 and BiP from the IRE1α/BiP/ERDJ4 complex, the IRE1α signaling pathway is initiated causing splicing of Xbp1 mRNA and its translation into XBP1 protein, which travels to the nucleus to activate transcription of chaperones, such as ERdj4

Protein and ionic homeostasis in the endoplasmic reticulum (ER) are well balanced and essential for cellular survival. Disturbance of ER homeostasis induces signalling cascades by activating three different transmembrane proteins (IRE1α, PERK, and ATF6), all three of them being located in the ER membrane. IRE1α (inositol-requiring enzyme 1 alpha) is a transmembrane kinase, which also possesses endoribonuclease activity [1] (Fig. 1). In an inactive state, the luminal domain of IRE1α is bound to BiP, the central ER-resident chaperone, which belongs to the Hsp70 protein family. Unfolded or misfolded proteins within the ER require BiP for their correct (re-) folding. When increasing amounts of misfolded proteins accumulate in the ER, BiP dissociates from IRE1α, thereby possibly enabling unfolded protein within the ER to bind to IRE1α [2]. This leads to autophosphorylation of IRE1α, and activation of the cytosolically located endoribonuclease domain [3]. Activated, phosphorylated endoribonuclease splices the prevailing mRNA of X-box-binding protein 1 (Xbp1), generating spliced Xbp1 mRNA (Xbp1s). Xbp1s is efficiently translated into XBP1 protein, a transcription factor that travels to the nucleus to induce the transcription of a variety of genes associated with the ER-associated degradation (ERAD) pathway, as well as the folding machinery in the ER lumen [4, 5]. The second transmembrane protein that is activated by ER stress is PERK (protein kinase RNA-like ER kinase), which is also silenced by its binding to BiP. Activation of PERK leads to phosphorylation of elongation initiation factor 2α (eIF2α) [1], which inhibits general protein translation in the cytosol, but simultaneously stimulates preferential translation of a subset of proteins, including ATF4 (activating transcription factor 4). ATF4 increases transcription of chaperones to upregulate chaperone levels within the ER to restore ER homeostasis. The third ER-stress-mediating transmembrane protein is ATF6 (activating transcription factor 6). As IRE1α and PERK, ATF6 is also inactivated by its binding to BiP. Dissociation of BiP from ATF6 results in trafficking of ATF6 to the Golgi complex, where the N-terminal, cytosolic part of ATF6 is cleaved and travels to the nucleus to induce transcriptional activation of BiP and other chaperones. ATF6 also induces ERAD components when heterodimerized with the transcription factor XBP1 [6]. If ER homeostasis cannot be restored by these signalling cascades, apoptosis is induced at late stages of ER stress.

As a member of the Hsp70 family, BiP has an intrinsic ATPase activity, which is essential for the energy-consuming folding process of target proteins. This ATPase activity is stimulated by co-chaperones, a protein family characterized by the highly conserved J-domain structure, which is necessary for binding to BiP and for activating its ATPase activity. So far, seven J-domain proteins (ERDJ1—ERDJ7) are known to be localized in the ER. Two of them (ERDJ1 and ERDJ2) have been shown to have similar stress-sensing and cell-protective functions as the classical transmembrane proteins mentioned above. ERDJ1 and ERDJ2 both bind to BiP, and in the BiP-bound complex, they permit co-translational translocation of proteins. When released from BiP, both ERDJ1 and the ERDJ2/SEC62 complex block co-translational translocation by binding near the tunnel exit of the large ribosomal subunit (see review [7]).

In this review, we wish to focus on ERDJ4, a co-chaperone originally discovered in 2001 [8]. Increasing amounts of data have since been published. While most (co-)chaperones are found throughout all species, ERDJ4 only seems to exist in vertebrates, thus suggesting that it has specialized during evolution. To understand the partly controversial data, we closely re-examined and analyzed all original data published so far to achieve a comprehensive picture of the localization and function of ERDJ4.

### Structure and subcellular localization

ERdj4 [9], also known as Mdg1 [8], Mdj7 [10], and DNAJB9 [11], encodes a protein that consists of 222 aa and has an estimated size of 25.7 kDa [8]. At its N-terminal end is a signal sequence (aa1-aa23) that targets ERDJ4 to the ER compartment. The J-domain (aa24-93) is located directly next to the signal sequence, which is followed by a glycine/phenylalanine rich region (G/F-rich) (aa 94–181). This region has been shown to be essential for interaction with target proteins such as RICTOR, a protein of the complex mTORC2 [12], the mutant surfactant protein C [13], and for the interaction and attenuation of IRE1α [14]. Sequence analysis further suggests that ERDJ4 contains two potential glycosylation sites [9]. However, endoglycosidase H digestion, which cleaves oligosaccharides from glycoproteins, as well as tunicamycin treatment, which inhibits protein glycosylation, revealed that ERDJ4 is not glycosylated [9, 15]. The C-terminus of ERDJ4 has a CSGQ sequence which—as a CAAX motif—might be a target for prenylation, a mode to anchor proteins to cellular membranes [16]. Whether the cytosolic pool of ERDJ4 protein is possibly attached to, or anchored at a membrane via this CAAX motif has not yet been investigated.

**Fig. 2:**
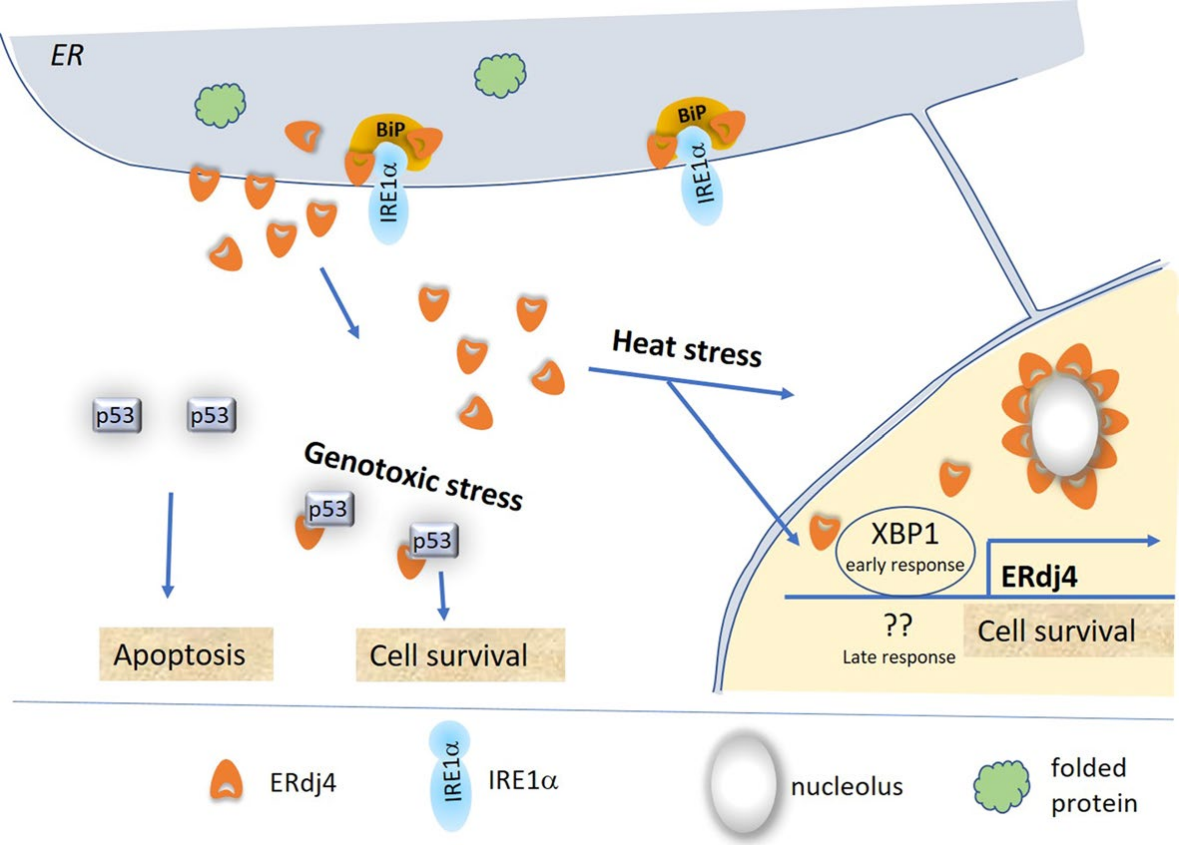
Schematic illustration of the ERDJ4-mediated response to heat and genotoxic stress. In genotoxically stressed cells and in inflamed cells, ERDJ4 binds to p53 to inhibit p53-induced apoptosis. In heat stressed cells, ERDJ4 travels to the nucleus, where it associates with nucleoli. In heat stressed cells, an early and a late stress response is induced, in which ERdj4 mRNA levels are upregulated. The early response is thought to be mediated by the transcription factor XBP1. The transcription factors controlling the late response have not been identified yet

ERDJ4 was originally reported to be exclusively located in the ER lumen [9, 17]. It has now become increasingly clear that different protein pools of ERDJ4 exist, an ER-luminal and a cytosolic pool. Under control conditions, about 70–75% of the protein resides in the ER lumen, while 25—30% resides as an integral membrane protein at the ER membrane facing the cytosol [9, 12]; see also a recent review [7]. Specific stressors, such as heat shock, genotoxic stress, but also inflammation, direct the entire protein pool to the nucleus [8, 18–20] (Fig. 2). In heat-shocked cells, ERDJ4 protein localizes to the nucleoli; the translocation is reversed upon recovery from heat shock [8, 18]. This translocation to the nucleus does not occur when cells are treated with other stressors such as tunicamycin or shear stress [18]. It is of interest that also in chronic wound tissue, ERDJ4 protein is localized in the nuclear compartment of lymphocytes and granulocytes, as was shown by immunohistochemical stainings [20]. In fibrillary glomerulonephritis, substantial amounts of ERDJ4 protein are deposited extracellularly and can be detected in blood samples [21–24]. In summary, ERDJ4 is located in the ER lumen, at the cytosolic side of the ER membrane, in the nucleus, and even in the extracellular space and in blood.

### Regulation of ERdj4

ERdj4 mRNA levels are very low under control conditions and are upregulated manifold by different chemical ER stressors, such as tunicamycin, thapsigargin, ionomycin, DTT, NO, methanol, or ethanol [8, 15, 18], but ERdj4 levels are also elevated by misfolded proteins, such as the aggregation-prone amyloid beta protein [25], mutant surfactant protein C, and mutant insulin [13] (see also Figs. 1 and 2).

Except for COS-7 and T293 cells, ERdj4 expression levels are elevated in all cell lines examined in response to stress—though to different extents [8]. Tunicamycin treatment not only upregulates ERdj4 transcription, but also increases ERdj4 mRNA stability [18]. Notably, induction of ERdj4 expression is strongest in endothelial cells compared to non-endothelial cell types, with the highest induction (36-fold) in tunicamycin-stressed endothelial cells [18]. All stressors mentioned above have a common theme, which is the diminished availability of BiP due to increased amounts of misfolded protein in the ER. In accordance with these findings, direct downregulation of BiP by siRNA also elevates the mRNA levels of ERdj4 [26, 27], thus confirming that BiP-controlled signaling pathways upregulate ERdj4 transcription. During ER stress induced by tunicamycin, thapsigargin, or LPS, XBP1 seems to be the most effective transcription factor for upregulating ERdj4 transcription [28, 29]. XBP1 drives ERdj4 transcription by its binding to the ACGT-binding site within the ERdj4 promoter [28]. Cotransfection of HEK-cDNA5 cells with an ERdj4 promoter construct, driving the luciferase gene as reporter, along with either XBP1, ATF4, or ATF6, showed that the ERdj4 promoter can be activated by all three transcription factors, most efficiently by XBP1 and less efficiently by ATF4 [30]. Genotoxic agents such as doxorubicin, actinomycin D, etoposide, or mitomycin c were also identified as inducers of ERdj4 transcription. Interestingly, these agents only upregulate ERdj4 expression in the presence of p53 [19]. Experimental data using different inhibitors revealed that the p53-dependent upregulation of ERdj4 is mediated via the Ras/Raf/ERK pathway [19].

Besides the classical ER stressors and genotoxic agents, ERdj4 is also induced by heat stress [8, 18, 30, 31] (see Fig. 2), with an early transient response, as shown in microvascular endothelial and HeLa cells [18], and a delayed prominent increase in the recovery period, as shown in HEK-cDNA5 cells [30]. Since heat stress has been shown to induce splicing of Xbp1 [30], the initial heat shock-induced transcriptional upregulation of ERdj4 could well be mediated by XBP1, while the delayed response (after an 18-h recovery period) is induced by yet unknown factors. It has been shown to be independent of XBP1, but also independent of ATF6, ATF4, or the classical heat shock transcription factor HSF1 [30].

Besides these cellular stressors, ERdj4 has also been shown to be induced by feeding, and it plays a central role in regulating the cellular metabolism, as outlined below [12].

### Metabolic function of luminal and cytosolic ERDJ4

A series of experiments were performed using whole-body constructs, as well as liver-specific constructs to dissect the functions of ERDJ4 [12]. Long-term ERDJ4 deficiency strongly induced the lipogenic transcription factor SREBP1c and resulted in a substantial increase in fat body mass with a corresponding loss of lean body mass [12]. Conversely, ERdj4 overexpression reduced lipid accumulation and increased liver mass, possibly due to increased protein synthesis [12]. Profound experimental studies revealed that the luminally located ERDJ4 protein is largely involved in controlling lipogenesis. When re-expressing ERdj4 in ERdj4 knockout cells, ERDJ4 was shown to mediate the degradation of SREBP1c protein [12]. This ERDJ4-induced degradation of SREBP1c can, at least partially, be rescued by addition of the ERAD inhibitor NMS-873, pointing to the degradation of SREBP1c via the ERAD pathway [12]. Further experiments were performed to elucidate the function of the cytosolic ERDJ4 protein pool using a truncated ERdj4 construct that was devoid of the signal sequence. Co-immunoprecipitation experiments with these truncated ERDJ4 proteins identified the complex mTORC2 and components of mTORC2, RICTOR and mTOR, as novel cytosolic clients of ERDJ4 [12]. Binding of ERDJ4 to mTORC2 was measured in about one-third of the mTORC2 pool. This ERDJ4/mTORC2 complex exhibited downregulated kinase activity, suggesting that ERDJ4-bound mTORC2 keeps the complex in a silenced state [12]. In promoting complex formation of mTORC2, and by surveying its kinase activity, ERDJ4 controls protein synthesis and improves insulin signaling, both pathways being largely affected in ERdj4-deficient cells [12].

These data reveal that ERDJ4 holds a central position in the cellular metabolism. It has not yet been investigated whether this metabolic function of the cytosolic ERDJ4 requires the J-domain or is J-domain-independent. Since the ability of ERDJ4 to stimulate the ATPase activity of HSP70 chaperones is not restricted to the ER-resident BiP, as was shown in in vivo complementation studies in *E. coli* [8], the folding of mTORC2 components could be mediated by ERDJ4/HSP70. In any case, the ability of co-chaperones to form dimers or multimers is relevant to the discussion on the mediation of the assembly of the protein complex mTORC2.

### Antiapoptotic function of ERDJ4

In stressed cells, ERDJ4 has a pro-survival effect. This was shown in various experimental approaches using either the human neuroblastoma cell line SK-N-SH or human stem cells (HSCs) [15, 32]. Stressing of the cells was performed by application of tunicamycin, which inhibits glycosylation of proteins, thereby specifically inducing the accumulation of misfolded proteins in the ER. In SK-N-SH cells, overexpression of ERdj4 reduced cell death in response to tunicamycin treatment, an observation based on time-course studies of cell death in transfected cells, as well as on measurements of LDH activity in the media [15]. It was further shown that the deletion of the J-domain of ERDJ4 abolishes the anti-apoptotic effect, indicating that ERDJ4–BiP interaction is crucial for the anti-apoptotic function of ERDJ4 [15]. Similar results were obtained in HSC, in which tunicamycin treatment induced cell death via the PERK/eIF2alpha/ATF4/CHOP/GADD34 pathway [32]. Strong ERdj4 overexpression in HSC could protect HSCs from tunicamycin-induced apoptosis. Even subtle overexpression of ERdj4 could prevent CHOP and GADD34 induction in HSCs after transplantation, thereby promoting survival of the cells [32]. This is of clinical relevance, since overexpression of ERdj4 was shown to improve engraftment of transplanted HSCs [32]. Furthermore, ERDJ4 was shown to be necessary for the survival of precursor B cells [33]. In mice with a disrupted ERdj4 gene locus (ERdj4^GT/GT^), analyses of the B-cell pool revealed a reduced number of large and small pre-B cells, and of immature B cells due to increased rates of apoptosis at the pro-B-cell stage [33]. The underlying molecular mechanism for promoting cellular survival could be based on the interaction of ERDJ4 with the tumor suppressor p53, as ERDJ4 was shown to inhibit the pro-apoptotic signaling of p53 in response to genotoxic stress [19]. Co-immunoprecipitation experiments further showed that ERDJ4 binds to p53 in nuclear as well as cytoplasmic fractions of SK-N-SH and U2OS cells. ERdj4 mutants that lack the J-domain lose the ability to bind to p53 and also to inhibit the pro-apoptotic function of p53. These results can be interpreted in at least two ways: either that ERDJ4-p53 interaction depends on ERDJ4-BiP interaction via the J-domain, or that ERDJ4 directly binds to p53 via its J-domain [19]. In inhibiting apoptosis, ERDJ4 strongly promotes cellular survival of proB cells, which is essential for a functional immune response but also, in general, for the survival of stressed cells. Survival of stressed cells is not always beneficial to the entire organism when considering, for example, the growth of tumor cells and their metastasizing potential. Yet tumor cell growth is always closely linked to dedifferentiation of cells, a process that also seems to be controlled by ERDJ4, as outlined in the next section.

### Epithelial–mesenchymal transition and differentiation are controlled by ERDJ4

ERDJ4 has been shown to play a pivotal role during differentiation processes. Being originally discovered in differentiating rat endothelial cells, it was suspected to play a role in angiogenesis [8]. In fact, ERdj4 expression peaks at a stage when migration and proliferation of endothelial cells ceases and the remodeling process is initiated [18]. Microarray analyses have further revealed that mesenchymal stem cells, which have the potential to differentiate into endothelial cells, also express—among other endothelial differentiation specific genes—ERdj4, which, it has been suggested, confers endothelial differentiation potential to these cells [34]. Further evidence for its role in differentiation processes came from immunohistochemical studies performed during early embryonic development. While in mesenchymal cells the ERDJ4 protein revealed a salt and pepper pattern, ERDJ4 protein marks apical and/or basal compartments in epithelial linings, which is thought to prevent mesenchymalization of these cells [35]. In this context, the ERDJ4 protein might either control folding and secretion of membrane-bound receptors or inhibit the IRE1α/XBP1/SNAI1/2-induced mesenchymalization [14, 36]. Inhibition of the IRE1α signalling pathway could be mediated by binding of ERDJ4 to IRE1α, which was shown to promote heterodimerization of BiP/IRE1α, thereby inhibiting the IRE1α-induced signalling cascades [14] (Fig. 1). Recently, the role of ERDJ4 in inhibiting epithelial–mesenchymal transition (EMT) was confirmed and analyzed in detail in triple-negative breast cancer [37]. Experimental downregulation of ERDJ4 resulted in a significant downregulation of the epithelial marker E-cadherin, upregulation of the mesenchymal marker vimentin, and increased nuclear translocation of ZEB1. Conversely, overexpression of ERDJ4 resulted in downregulation of vimentin, reduced nuclear localization of ZEB1, and significantly inhibited the metastatic colonization of breast cancer cells in the lung [37]. The role of ERDJ4 in inhibiting epithelial–mesenchymal transition has also been confirmed by another study, which showed that high ERDJ4 levels lower the metastatic ability of tumor cells [38]. Also in retinal cells, where the transition of endothelial-to-mesenchymal phenotype is a key-cellular phenomenon during diabetic retinopathy, ERDJ4 is suspected to play a central role. High glucose levels were shown to downregulate the long non-coding RNA H19 (lncRNA H19), while its overexpression prevented EMT [39]. As a target of the lncRNA H19 [40], it is reasonable to speculate that ERDJ4 also controls EMT in diabetic retinopathy.

Besides the function of ERDJ4 in controlling EMT, ERdj4 also has been shown to be an important regulator of the functional immune response. Using gene-trapped mice (ERdj4^GT/GT^) with a disrupted ERdj4 gene locus, ERDJ4 was shown to control B-cell differentiation and genomic switching of immunoglobins [33]. Besides the function of ERDJ4 in promoting the survival of precursor B cells (see previous section), serum levels of IgG, IgE, and IgA were increased in ERdj4^GT/GT^ mice, while the IgM levels remained unaffected. This finding suggests that ERDJ4 suppresses immunoglobulin class switching in healthy mice [33]. All these experimental data point to the central role of ERDJ4 in regulating differentiation processes by controlling EMT and—in case of B-cell differentiation—genomic switching.

### ERDJ4 mediates folding and degradation of target proteins in highly secretory cells

ERdj4 expression levels were examined in human, rat, and mouse tissue. ERdj4 was shown to be expressed in most human tissues, with the highest expression in the tissues of the placenta, liver, kidney, prostate, testis, ovary, lung, in the small intestine, and colon, all of which are highly secretory active tissues [8, 9, 15]. High expression in secretory active cells was confirmed by in situ hybridization studies of adult human placenta, stomach, and lung, a technique that allows visualization of the regional expression of ERdj4 mRNA [8]. In human placenta, ERdj4 expression was high in endothelial cells, as well as in trophoblast cells, in the human gut in epithelial, stromal, and lymphatic cells, and in the respiratory tract in nasal epithelial cells [41], in bronchial epithelium, alveolar macrophages and type II pneumocytes [8]. These mRNA patterns were further confirmed by immunohistochemical studies showing high ERDJ protein levels in secretory active cell types, such as ependymal cells or enterocytes [35]. Additional evidence for the central role of ERDJ4 in secretory active tissue came from transgenic mice with defective ERDJ4 [42]. Besides their reduced body and liver weight at E18.5, secretory active cells and tissues, such as fibroblasts, lung tissue, kidney, salivary gland, and pancreas tissue experienced constitutive ER stress. In pancreatic β cells, this stress was associated with β cell loss, hypoinsulinemia, and glucose intolerance, probably as a consequence of impaired insulin maturation. In these ERdj4^GT/GT^ mice, increased levels of plasma proinsulin, as well as increased proinsulin:insulin ratios, were measured, pointing to a “chaperoning” activity of ERDJ4 in mediating proper folding and maturation of proteins.

In the cells of the respiratory tract, chaperoning and “over-chaperoning” activity was reported for ERDJ4 when mediating the folding of the cystic fibrosis transmembrane conductive regulator (CFTR) and the mutant ∆F508 CFTR protein. Here, downregulation of ERdj4 by siRNA increased both WT-CFTR and mutant ∆F508 CFTR protein levels intracellularly, as well as at the plasma membrane [41]. Conversely, overexpression of ERdj4 increased levels of ubiquitinated WT-CFTR and mutant ∆F508 CFTR in cells, thus suggesting that ERDJ4 promotes ER-associated degradation (ERAD) of CFTR under physiological conditions, and that downregulation of ERdj4 ameliorates cystic fibrosis disease by decreasing the degradation rate of CFTR and of mutant ∆F508 CFTR, as shown in mouse intestine [41]. The targeting of proteins to the ERAD pathway has also been shown in experiments using Xenopus oocytes. When co-injecting ERdj4 and the ENaC channel, the current through the channel, as well as its surface expression, is reduced [43]. Treatment with proteasome inhibitors prevents ENaC current reduction by ERDJ4, strongly indicating that ERDJ4 promotes ENaC degradation via proteasomal degradation [43].

Alveolar type II pneumocytes secrete surfactant protein C, which is essential for lowering the surface tension in the lung. In contrast to WT-surfactant protein C, the turnover of which is only slightly affected by knockdown of ERdj4, mutant surfactant protein C is another candidate that has been shown to be targeted for its degradation by ERDJ4. This degradation pathway is mediated in concert with BiP and ERDJ5 [13]. Misfolded surfactant protein C and overexpressed ERdj4 were shown to coprecipitate with p97/VCP, indicating that ERDJ4 (together with ERDJ5) remains associated with the misfolded proprotein until it is dislocated to the cytosol [13]. This ERDJ4-mediated degradation pathway is at least active in stressed cells, in which high levels of ERdj4 are prevailing [13]. Under non-stressed control conditions, ERdj4 levels are very low [8, 9] and the co-IP experiment failed to show interaction of ERDJ4 and p97/VCP [17]. Altogether, the experimental data reveal that under stress conditions—when ERdj4 levels are high—ERDJ4 and p97/VCP bind at both sides of the ER membrane to client proteins destined for degradation. A close association of ERDJ4 with the retrotranslocon has been reported, even under control conditions, when ERdj4 levels are low. This is shown by co-IP experiments in which ERDJ4 coprecipitated with Derlin1 [17], an integral ER membrane protein shown to form a protein channel for translocation of misfolded proteins [44].

The degradation pathways require two steps: binding to the target protein and its delivery to the ERAD pathway. The binding to target proteins seems to necessitate specific sequences within target proteins that are recognized and bound by ERDJ4. By screening a peptide library of a non-secreted mouse kappa antibody light chain (NS1 LC) and of a truncated version of a mouse gamma 1 antibody heavy chain (mHC), these sequences could be identified and were shown to be more specific than those required for binding of BiP [45]. Computational analysis identified the sequences ERDJ4 binds to as aggregation-prone. Disruption of these binding sites was able to extend the half-life of the mutated peptides, suggesting that, in fact, ERDJ4-binding primes peptides to their degradation [45]. While binding of target protein is J-domain-independent, the delivery to the ERAD pathway requires a functional J-domain, as was shown, for example, for the degradation-prone mutant surfactant protein C [13]. Furthermore, binding of ERDJ4 to surfactant protein C was strengthened when the J-domain was mutated [13]. Enhanced binding of ERDJ4 J-domain mutants to light chains was also reported in U2O2 cells [17]. Accordingly, ERDJ4 recognizes and binds to specific sequences within target proteins in a J-domain-independent way, while targeting of the proteins to the degradation pathway is J-domain-dependent.

### Role of ERDJ4 in glucose metabolism

Glucose metabolism is controlled by glucagon and insulin, both of which are synthesized and secreted from pancreatic α and β cells, respectively. In the liver, glucagon initiates degradation of glycogen to generate glucose, which is released into the blood stream to increase plasma glucose levels. Insulin, on the other hand, enables glucose to be taken up by cells, thereby lowering blood glucose levels. This delicate balance in glucose metabolism is largely disturbed in ERdj4-deficient mice. Newborn ERdj4-deficient mice are severely hypoglycemic, and liver weights and hepatic glycogen stores are significantly reduced [12, 33, 42]. These data suggest that impaired glycogen synthesis and/or mobilization cause hypoglycemia and subsequent mortality in neonatal ERdj4^GT/GT^ mice.

Investigation of the pancreatic tissue in ERdj4-deficient mice revealed a substantial increase in the number of glucagon-synthesizing α cells, possibly causing the depleted glycogen stores and reduced liver weights. Furthermore, the number of insulin-producing β cells is reduced in ERdj4-deficient mice [42]. Ultrastructural analyses of the pancreatic islands revealed an increase in translucent, immature insulin granules, indicating that maturation of insulin is impaired. Impaired insulin maturation presumably also causes the reported increase in plasma proinsulin levels and hypoinsulinemia in ERdj4-depleted mice [42]. On the other hand, degradation of mutant, but also of WT insulin, was reported in HEK293 cells when cotransfected with ERdj4 and mutant (insulin 2C96Y) or WT insulin 2. Both proteins, WT and mutant insulin 2, were shown to be part of a complex destined for degradation, a process slowed down by siRNA [13]. These data show that glucose metabolism is severely impaired in ERdj4-deficient mice due to impaired glycogen synthesis/mobilization on one hand, and impaired insulin maturation and insulin degradation on the other hand. Accordingly, ERDJ4 seems to tightly control the amount of mature insulin. Another important circuit in insulin-directed metabolism is the insulin responsiveness of the target cells, which is also reported to be controlled by ERDJ4. In obese mice, the hepatic expression levels of ERdj4 are low, and restoration of hepatic ERdj4 expression effectively improves insulin sensitivity [12]. Recently, a series of ERdj4 knockouts and ERdj4-overexpressing cells showed that it is the cytosolic variant of ERDJ4 which has the ability to improve insulin sensitivity and signaling [12]. As outlined above, silencing of ERdj4 resulted in a functional loss of mTORC2, a protein complex that integrates nutrients and growth signals and regulates growth rates and metabolism [46]. All these data point to a central role of ERDJ4 in glucose metabolism, since it controls every circuit: glycogen storage and release of glucose in liver cells, insulin maturation, degradation, and insulin responsiveness. ERDJ4 deficiency is therefore closely associated with a diabetic phenotype.

### J-domain-dependent and -independent function of ERDJ4 in generation and degradation of amyloid peptides

Proteolytic degradation of the amyloid precursor protein (APP) by β- and γ-secretases and the accumulation and aggregation of the amyloid peptides Aβ40 and Aβ42 are major determinants in Alzheimer’s disease (AD) pathogenesis [47]. It has been shown that a large set of proteases regulates the production and degradation of prevailing amyloid concentrations [25, 48]. In ERdj4/BiP overexpressing cells, the levels of immature APP (imAPP) decreased, while levels of mature APP (mAPP) increased [25]. Co-immunoprecipitation experiments showed that BiP directly interacts with imAPP, suggesting a co-chaperoning function of ERDJ4 to stimulate ATPase activity of BiP in a J-dependent manner to generate mAPP [25]. Besides this J-domain-dependent co-chaperoning function of ERDJ4, the experimental data further show that Aβ40 is specifically degraded, leaving Aβ42 levels untouched [25]. This degradation of Aβ40 largely depends on the intactness of the J-domain and is accelerated in the presence of BiP. Yet, a minor, though statistically significant portion of Aβ40 is degraded in a J-domain-independent way [25]. While J-domain-dependency could be due to J-domain-dependent folding of proteases involved in degradation of Aβ40, J-domain-independency is rather due to the ability of ERDJ4 to bind to and to target proteins to ERAD, or to mTORC-mediated endosomal degradation. Protease-dependent degradation of Aβ is known to be rather complex and to depend on the dynamic equilibrium between various compartments and pathways [48]. Further studies are required to elaborate the role of ERDJ4 in Aβ generation to further understand the underlying molecular mechanism of AD and to develop tools that might prevent the generation of protein aggregates known to be neurotoxic in Alzheimer’s diseased brains.

### Serum levels of ERDJ4 as a biomarker for fibrillary glomerulonephritis

It was first suggested in 2018 that ERDJ4 is a biomarker specific for fibrillary glomerulonephritis (FGN) [21], a finding confirmed by subsequent studies [22, 49]. Protein patterns of glomerular biopsies from patients suffering from different renal diseases were compared with healthy glomeruli by mass spectrometry. These investigations revealed abundance of ERDJ4 as a specific marker for glomeruli of FGN patients [21, 50]. Immunohistochemical and immunofluorescence experiments showed abundance of ERDJ4 in glomerular capillary walls, the glomerular basal membrane, and the mesangium [21, 50]. Immunofluorescence further showed colocalization of ERDJ4 with IgG in extraglomerular deposits [21, 24, 50]. Immuno-EM experiments detected ERDJ4 in fibrils of fibrillary glomerulonephritis glomeruli, identifying ERDJ4 as a component of the fibril material [24]. The observation that abundant ERDJ4 is specific for FGN and that ERDJ4 colocalizes with IgG led to the hypothesis that ERDJ4 might act as an auto-antigen [50]. In addition to the high ERDJ4 protein levels measured in glomeruli of FGN patients, ERDJ4 levels were also elevated in the serum and, as such, serve as a rapid and suitable diagnostic marker [22, 23, 49]. In this study, it is the first time that ERDJ4 has been reported to be located in the extracellular space. We assume that ERDJ4 in the ER compartment is engaged in solubilizing the profibrillar material and is finally secreted into extracellular space where it accumulates, an event that seems to be specific for FGN.

## Conclusion

ERDJ4 is a co-chaperone that plays central roles in diverse processes, some of which are of increasing clinical relevance, such as the role of ERDJ4 in controlling epithelial–mesenchymal transition, cellular survival, cell differentiation, immunoglobulin switching, and insulin metabolism. A second major function of ERDJ4 is the degradation of proteins by binding to and targeting them to the ERAD pathway. It is not known which parameters determine the function of ERDJ4: whether it mediates maturation, degradation, assembly, or disassembly of proteins. The manifold functions of ERDJ4 are presumably determined by the respective microenvironment of ERDJ4 and can be attributed to its residency in various subcellular compartments, such as the ER lumen, the cytosol, and the nucleus. Some of the functions require the J-domain, such as the folding and maturation of proteins, and pro-survival activity. J-domain independency is reported to be responsible for the binding to client proteins destined to degradation, such as mutant surfactant protein C, mutant light chains, or Aβ40. Whether the J-domain-dependent co-chaperoning activity of ERDJ4 is required for targeting SREBP1c to the ERAD pathway, or for its function in assembling and controlling the activity of the cytosolically located mTORC2, remains to be elucidated. Specific stressors, such as heat shock and genotoxic agents, but also inflammation, direct the entire protein pool to the nucleus. It would also be of great interest to investigate the functional relevance of ERDJ4 in the nuclear compartment.

## Data Availability

No original data are included in this review.
